# Nanocrystalline Iron Pyrophosphate-Regulated Amorphous Phosphate Overlayer for Enhancing Solar Water Oxidation

**DOI:** 10.1007/s40820-022-00955-w

**Published:** 2022-10-31

**Authors:** Chengkai Xia, Yuankai Li, Minyeong Je, Jaekyum Kim, Sung Min Cho, Chang Hyuck Choi, Heechae Choi, Tae-Hoon Kim, Jung Kyu Kim

**Affiliations:** 1 School of Chemical Engineering, Sungkyunkwan University (SKKU), 2066 Seobu-ro, Jangan-gu, Suwon, 16419 Republic of Korea; 2 Theoretical Materials and Chemistry Group, Institute of Inorganic Chemistry, University of Cologne, Greinstr. 6, 50939 Cologne, Germany; 3 Department of Chemistry, Pohang University of Science and Technology (POSTECH), Pohang, 37673 Republic of Korea; 4 Department of Materials Science and Engineering, Engineering Research Center, Chonnam National University, Gwangju, 61186 Republic of Korea

**Keywords:** Water oxidation, Photoelectrochemical cell, Metal pyrophosphate, Electrochemical surface treatment

## Abstract

A rational regulation of the solar water splitting reaction pathway by adjusting the surface composition and phase structure of catalysts is a substantial approach to ameliorate the sluggish reaction kinetics and improve the energy conversion efficiency. In this study, we demonstrate a nanocrystalline iron pyrophosphate (Fe_4_(P_2_O_7_)_3_, FePy)-regulated hybrid overlayer with amorphous iron phosphate (FePO_4_, FePi) on the surface of metal oxide nanostructure with boosted photoelectrochemical (PEC) water oxidation. By manipulating the facile electrochemical surface treatment followed by the phosphating process, nanocrystalline FePy is localized in the FePi amorphous overlayer to form a heterogeneous hybrid structure. The FePy-regulated hybrid overlayer (FePy@FePi) results in significantly enhanced PEC performance with long-term durability. Compared with the homogeneous FePi amorphous overlayer, FePy@FePi can improve the charge transfer efficiency more significantly, from 60% of FePi to 79% of FePy@FePi. Our density-functional theory calculations reveal that the coexistence of FePi and FePy phases on the surface of metal oxide results in much better oxygen evolution reaction kinetics, where the FePi was found to have a typical down-hill reaction for the conversion from OH* to O_2_, while FePy has a low free energy for the formation of OH*.

**Graphical abstract:**

**Supplementary Information:**

The online version contains supplementary material available at 10.1007/s40820-022-00955-w.

## Highlights

- Inducing a localized crystalline iron pyrophosphate in amorphous iron phosphate overlayer.
- Enhanced photoelectrochemical water oxidation activity with long-term durability.
- The heterogeneous hybrid structure overcoming the energy barrier in water oxidation.

**Supplementary Information:**

The online version contains supplementary material available at 10.1007/s40820-022-00955-w.

## Graphical abstract

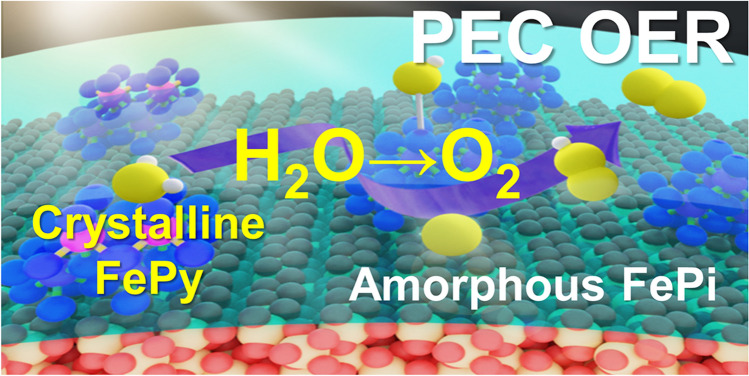

## Introduction

The demands for clean and sustainable energy continue to rise as fossil fuel reserves decline and environmental issues deepen, urging people to look for alternatives to reduce their carbon footprint [1–4]. Photoelectrochemical (PEC) solar water splitting is considered to be a promising alternative to produce clean H_2_ with high sustainability [5–7]. The challenges remaining to achieve high efficiency solar water splitting require the development of cost-effective and eco-friendly photoanode materials with enhanced PEC water oxidation performance by overcoming the typical sluggish oxygen evolution kinetics. Transition metal oxide (TMOs, ex. BiVO_4_, α-Fe_2_O_3_) [8–13] has been utilized for the PEC water oxidation because of its high chemical stability, earth-abundance. However, the poor electrical conductance and short minority carrier diffusion length always result in rapid recombination of photogenerated carriers and short-lived photogenerated holes [14–16]. The rapid recombination at surface states and sluggish reaction kinetics for the oxygen evolution reaction (OER) limit the PEC performance of pristine metal oxide photoanodes [17].

To overcome these limitations, surface modification by introducing co-catalysts or passivation layers is regarded as an effective strategy, which can ameliorate the surface states and improve reaction kinetics [18]. Transition metal phosphates (TMPs) have been widely used as the co-catalyst or passivation layer, where high OER activities have been reported in previous pioneering studies [19–23]. TMPs can effectively collect photogenerated holes from the bulk and suppress the rapid recombination of photogenerated holes at the surface states, thereby ameliorating the poor surface catalytic kinetics for pristine metal oxide [24–28]. However, these homogeneous metal phosphate overlayers were composed of a regular atomic configuration, which leads to unsatisfactory catalytic activity, poor intrinsic conductivity, and low chemical stability [29]. To address the drawbacks of homogeneous metal phosphates, introducing a novel hybrid structure with heterogeneous phosphate can be a promising approach. In general, metal phosphates have a variety of different crystal phases due to different compositions [30, 31]. In various metal phosphate families, a bulky pyrophosphate group has a distorted tetrahedral geometric structure, which can be easily formed with a high crystallinity and be conducive to obtaining high stability in alkaline media [32]. The distortion of the local geometric structure caused by the reorganization of the pyrophosphate ligand can contribute to favorable water adsorption [33]. Therefore, introducing pyrophosphate into the phosphate overlayer strategy can be a novel approach to achieve high efficiency solar water splitting with enhanced catalytic performance.

In this study, we combined nanocrystalline iron pyrophosphate (FePy) into an amorphous phosphate to form a novel hybrid overlayer (FePy@FePi), which significantly enhanced PEC water splitting performance of pristine TMOs photoanode. By harnessing a facile electrochemical treatment before phosphating, we performed the surface modification of the pristine α-Fe_2_O_3_ nanorods (NRs) with -OH anions. During the phosphating process, the electrochemically modified surface-induced phosphate-enriched regions, which resulted in localization of the nanocrystalline FePy phases in the overlaid amorphous iron phosphate (FePi) passivation layer on α-Fe_2_O_3_ NRs. Highly enhanced PEC water oxidation performance with long-term durability was obtained after decorating FePy@FePi, of which the current density value was 1.78 mA cm^−2^ in 1 M NaOH at 1.23 V versus reversible hydrogen electrode (RHE) under 1 sun illumination (100 mW cm^−2^). FePy@FePi hybrid overlayer improves the water oxidation performance of metal oxide photoanodes by 4.89-folds, which is far more effective than decorating with homogenous FePi overlayer (3.23-folds). The FePy@FePi hybrid overlayer dramatically enhanced the charge transfer efficiency. Moreover, the chemically stable FePy@FePi hybrid overlayer contributed to maintaining high PEC performance over 20 h of operation at 1.23 V versus RHE under 1 sun illumination. Density-functional theory (DFT) calculations illustrate the generation mechanism of the FePy@FePi hybrid overlayer and its reaction mechanism. The FePy phase has a down-hill free energy (− 2.86 eV) for water adsorption while the FePi phase has a typical down-hill reaction for the conversion from OH* to O_2_. The hybrid of FePy and FePi overcomes the bottleneck reaction steps in the water oxidation pathway through the intermediates exchange between FePy and FePi phases in the hybrid overlayer. This work provides a strategy to synthesize a hybrid heterogeneous phosphate overlayer, which is positive to improve the water oxidation activity of transition metal oxide photoanodes.

## Experimental Section

### Synthesis of FePy@FePi Decorated Photoanode

#### Synthesis of α-Fe_2_O_3_ Nanoarrays

All chemicals were analytically pure and are listed in the supporting information. The α-Fe_2_O_3_ nanoarrays were prepared on FTO glass substrates by a hydrothermal method. A Teflon-lined stainless autoclave (50 mL) was filled with 40 mL of an aqueous solution containing 0.1 M FeCl_3_·6H_2_O and 0.15 M (NH_2_)_2_CO before the pH was adjusted as 1.6 by a concentrated HCl solution. The FTO glass was thoroughly cleaned with isopropanol and deionized water. Then, the cleaned FTO glass slide was immersed vertically into the solution and heated at 110 °C for 6 h. The FeOOH film obtained on FTO was repeatedly cleaned by deionized water to remove impurities. Finally, the FeOOH was dried by N_2_ followed by annealing in air at 550 °C for 2 h and 800 °C for another 20 min to obtain the α-Fe_2_O_3_ nanoarrays.

#### Electrochemical Pretreatment

A three-electrode cell was employed with the α-Fe_2_O_3_ nanoarrays as the working electrode, Ag/AgCl as the reference electrode, and platinum electrode as the counter electrode. A constant potential of 1.2 V versus Ag/AgCl was applied in the aqueous electrolyte (pH 7) at room temperature (25 °C) for 30 min.

#### Phosphating Treatment

In order to synthesis FePy@FePi hybrid overlayer, 100 mg of NaH_2_PO_2_·H_2_O crystalline powder and the electrochemically treated α-Fe_2_O_3_ nanoarrays were placed at the two separate positions of the tube furnace while the NaH_2_PO_2_·H_2_O was near the Ar inlet. The tube furnace was heated in a high-purity Ar atmosphere to 300 °C at a slow heating rate of 2 °C min^−1^, maintained at this temperature for 2 h, and then naturally cooled down to room temperature. For FePi, the same tube furnace-based experimental protocol was applied by using pristine α-Fe_2_O_3_ nanoarrays.

### Characterization

The X-ray diffraction (XRD) characterization was carried out using an X-ray diffractometer (Bruker D8 Discover, Germany) at 40 kV with Cu Kα radiation. X-ray photoelectron spectroscopy (XPS) measurements were taken by ESCALAB 250 (Thermo Fisher Scientific), where the X-ray source was AI Kα. Scanning electron microscopy (SEM) images were obtained by a JEM-3010 field emission scanning electron microscope (JEOL, Japan). High resolution transmission electron microscopy (HR-TEM) images and energy-dispersive X-ray spectroscopy (EDS) elemental maps were acquired using a JEM ARM 200F transmission electron microscope (JEOL, Japan).

### Photoelectrochemical Characterization

All PEC characterizations were performed on an electrochemical workstation (Reference 600 + , Gamry) using a three-electrode system. The as-prepared photoanodes (the active area was determined by using an aperture with an exposed area of 0.258 cm^2^) served as the working electrode, while Hg/HgO (1 M NaOH solution) and Pt wire were the reference electrode and counter electrode, respectively. Linear sweep voltammetry (LSV) curves of the photoanode were recorded at 10 mV s^−1^ under an AM 1.5G simulated 1 sun irradiation (150 W xenon lamp, PEC-L01, PEC Cell, Yokohama, Japan) with a power intensity of 100 mW cm^−2^. Unless otherwise specified, all of the PEC tests were illuminated from the back-side and all the potentials were normalized to the reversible hydrogen electrode potential (RHE, *E*_RHE_ = *E*_Hg/HgO_ + 0.059pH + 0.140 V). The electrochemical impedance spectroscopy (EIS) was recorded in the range of 100,000–0.01 Hz at 1.23 V versus RHE with an amplitude of 5 mV under simulated 1 sun irradiation. To determine *R*_ct_ and *R*_tr_, all of the Nyquist plots were normalized by a series resistance value of ~ 28 Ω. Mott–Schottky measurements were taken at a scan rate of 10 mV s^−1^ and a frequency of 1000 Hz in a 1 M NaOH solution (pH = 13.6). Therefore, the Mott–Schottky equation can be used to determine the donor concentration (*N*_d_) and flat band potential (*V*_fb_). The charge separation effect (*η*) was determined by LSV measurements in 1 M NaOH solution containing 0.5 M Na_2_SO_3_ as a hole scavenger.

## Results and Discussion

### Characterization of FePy@FePi Hybrid Overlayer

As substrate materials, pristine α-Fe_2_O_3_ NRs were fabricated on the surface of a FTO glass by using a simple hydrothermal synthesis method [34]. To prevent side effects or unexpected reactions, no additional dopants such as Ti were used in this study. For the synthesis of FePy@FePi hybrid overlayer, chemical vapor deposition (CVD)-mediated surface phosphating was conducted after the electrochemical surface pretreatment on the surface of α-Fe_2_O_3_ NRs under 1.2 V versus Ag/AgCl at a mild pH of 7 for 30 min. As a comparison, a conventional FePi overlayer was also introduced on the same substrate material by using the same CVD-mediated surface phosphate without any electrochemical surface pretreatment. Detail experimental procedure is given in supporting information.

**Fig. 1:**
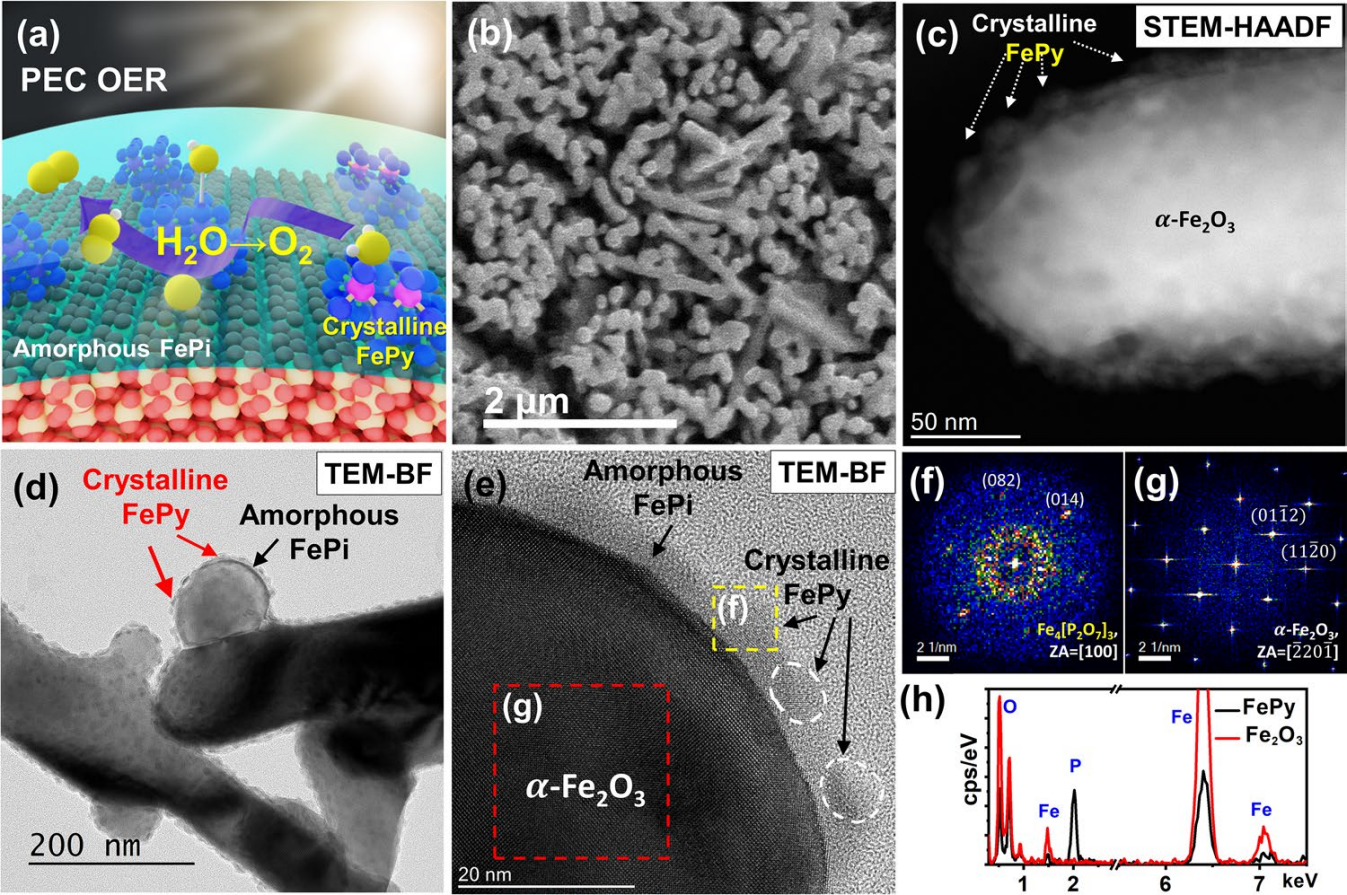
**a** Schematic illustration.** b** SEM image.** c** HAADF-STEM image. **d, e** TEM-BF images of FePy@FePi decorated nanorods. **f, g** FFT obtained from **e**.** h** EDS elemental spectra obtained from **f** and **g**

As shown in Fig. 1a-b, the schematic illustration and SEM images of the FePy@FePi hybrid overlayer decorated α-Fe_2_O_3_ nanorods, respectively. In Fig. 1b, the decorated α-Fe_2_O_3_ photoanode has a vertical nanorod array morphology and the length of the NRs was around 500 nm (Fig. S1). The high angle annular dark field (HAADF) scanning TEM (STEM) image of FePy@FePi hybrid overlayer (Fig. 1c) clearly shows dark and bright regions and the TEM bright field (BF) image also shows a very significant difference in the overlayer (Fig. 1d). The HR-TEM image shows that FePy@FePi hybrid overlayer has amorphous structure, and there are some dark regions in the amorphous layer (Fig. 1e). Moreover, the HRTEM images in Fig. 1e clearly show a series of lattice fringe (Fig. S2) with *d* = 0.256, 0.224, 0.238, and 0.145 nm, corresponding to the (072), (014), (004), and (400) plane diffraction of Fe_4_(P_2_O_7_)_3_, respectively. After further magnifying the interface of the overlayer and bulk, it can be seen that the dark regions in the overlayer are separated nanocrystalline phases. To further confirm the structural features of the nanocrystalline phases, TEM and EDS studies were performed to provide direct evidence for the atomic-scale arrangement of the elements. The fast Fourier transform (FFT) of the matches to the crystalline phases in the overlayer and bulk are shown in Fig. 1f-g, respectively. The crystalline phases in the overlayer are expected to be iron pyrophosphate (Fe_4_(P_2_O_7_)_3_) and the bulk region can be confirmed to be α-Fe_2_O_3_ because the FFT image provides *Z*-contrast, which is atomic number dependent. In addition, based on the element ratios provided by the EDS spectrum **(**Fig. 1h), it can be confirmed that pyrophosphate compounds (P_2_O_7_) exist in the nanocrystalline phase of the overlayer. EDX images of the FePy@FePi hybrid overlayer (Fig. S3) show the heterogeneous distribution of Fe, O, and P. Correspondingly, in the HR-TEM image of FePi overlayer decorated metal oxide nanorods (Fig. S4), there is a uniform amorphous overlayer on the surface of nanorods structure, while it does not show any crystal structure similar to that in FePy@FePi hybrid overlayer.

**Fig. 2:**
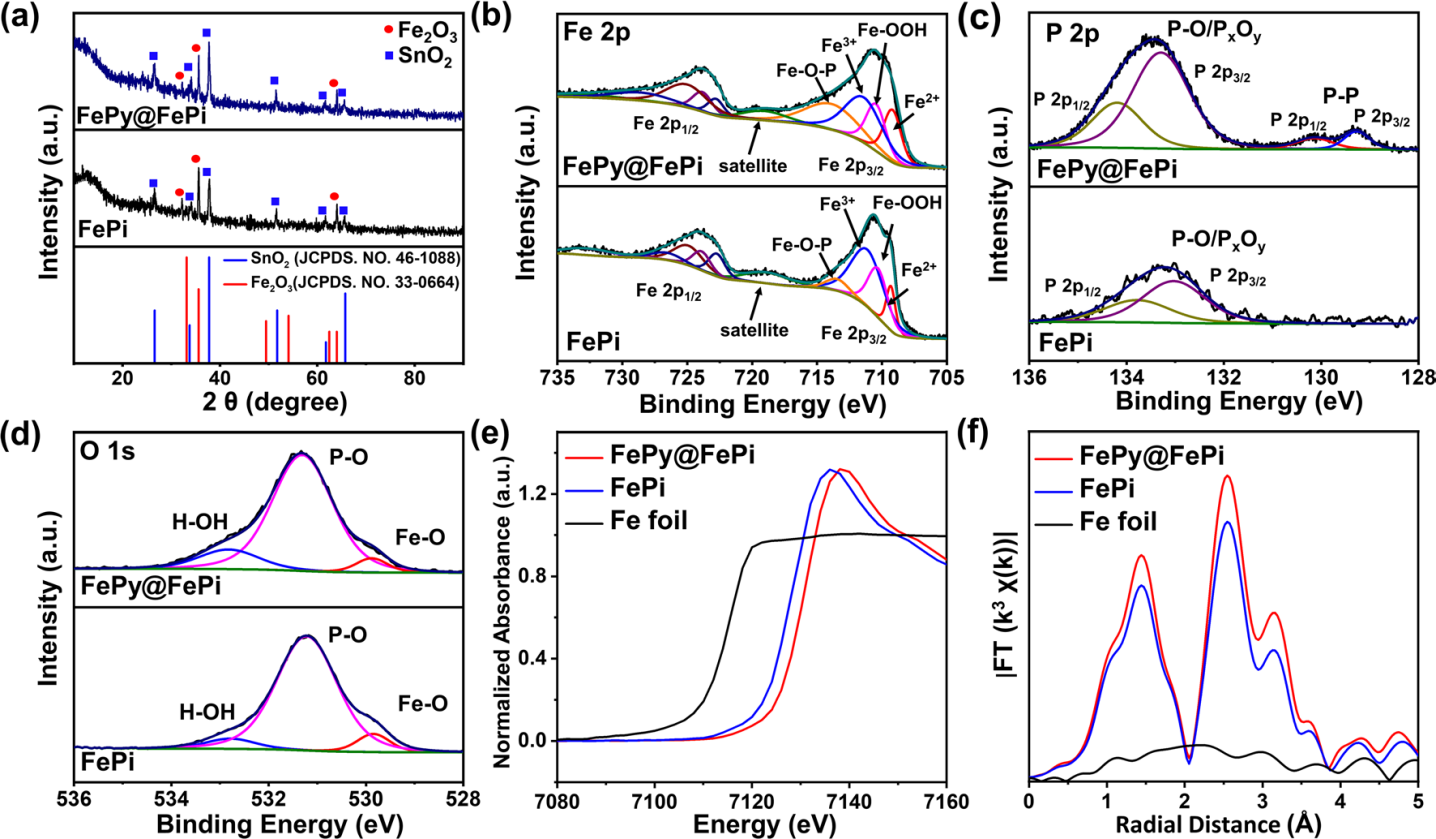
**a** XRD patterns of FePi and FePy@FePi. **b, c, and d** XPS spectra of Fe 2*p,* O 1*s*, and P 2*p* of FePi, and FePy@FePi. **e, f** XANES and EXAFS spectra of Fe foil FePi, and FePy@FePi

Figure 2 shows the XRD patterns and XPS spectra of FePi and FePy@FePi decorated samples. All of the XPS data were calibrated with the C 1*s* spectrum where the binding energy of major C–C peaks is 284.6 eV (Fig. S5). The characterization results for pristine α-Fe_2_O_3_ and electrochemically treated α-Fe_2_O_3_ (e-Fe_2_O_3_) are shown in Fig. S6. The XRD patterns (Fig. 2a) show similar diffraction peaks indexed to SnO_2_ of the FTO substrate (JCPDS No. 46–1088) and α-Fe_2_O_3_ (JCPDS No. 33–0664) [35]. There are no diffraction peaks for any iron phosphates compounds because the overlayer is too thin to get reliable result. The pattern of e-Fe_2_O_3_ (Fig. S6a) shows a diffraction peak at 64.33, which is related to FeOOH species, indicating surface oxidation after the electrochemical pretreatment. Figures 2b and S6b show the high resolution XPS spectrum of Fe 2*p*. The deconvoluted peaks for Fe 2*p*_1/2_ and Fe 2*p*_3/2_ at binding energies (BE) of around 726.2 and 712.2 eV, respectively, confirm the existence of Fe^3+^ in these four photoanodes. The deconvoluted peaks at BEs of 723.6 and 710.0 eV confirm the existence of Fe^2+^ [36, 37]. The deconvoluted peaks at BE values around 724.8 and 711.0 eV correspond to FeOOH species. Importantly, in the spectra of FePi and FePy@FePi, another pair of peaks which correspond to the Fe–O-P bond appeared at 714.4 and 728.2 eV, which indicates that the phosphating treatment formed an ultrathin or amorphous iron (pyro)phosphate layer [38]. In Fig. 2c, the peaks centered at ca. 133.5 eV, which is assigned to the P-O bond in the pyro/phosphate ion (P_x_O_y_), were observed in FePi and FePy@FePi. The slight positive shift was observed in the two deconvoluted peaks centered at 133.3 and 134.2 eV for FePy@FePi comparing to those of FePi, attributed to the different PO bonds in PO_4_^3−^ and P_2_O_7_^4−^. Deconvoluted peaks centered at 129.2 eV (P 2*p*_1/2_) and 130.1 eV (P 2*p*_3/2_) for the P-P bond were observed in FePy@FePi [30, 38–40]. Since no peaks related to Fe–P bonds appear in the Fe 2*p* spectrum, these P-P bonds were attributed to phosphorus species generated during the phosphating process [41]. This was further confirmed by the O 1*s* spectra in Fig. 2d. The O 1*s* XPS spectrum of FePi and FePy@FePy can be deconvoluted into three peaks, where the lower BE at 529.9 eV and higher BEs at 531.5 and 532.85 eV corresponding to the Fe–O bond, P-O bond, and surface-absorbed hydroxyl group (H_2_O), respectively [42, 43]. The O 1*s* spectrum of FePy@FePi can be deconvoluted into four peaks and the peaks at 531.3 and 531.7 eV correspond to Fe–O-P and P-O-P bonds, which confirms that two different phosphates appeared on the surface of the sample after electrochemical pretreatment and phosphating treatment [40]. In addition, by integrating the XPS spectrum of e-Fe_2_O_3_, it can be found that FeOOH is produced on the surface of α-Fe_2_O_3_ NRs after the electrochemical treatment. The relatively enriched regions of FeOOH will be favorable for the absorption of phosphide species, thereby affecting the phosphating treatment.

The difference in electronic structure and local chemical configurations of FePy@FePi and FePi were studied by X-ray absorption near edge structure (XANES) and extend X-ray absorption fine structure (EXAFS). Figure 2e shows the Fe K-edge XANES spectra of FePy@FePi, FePi, and Fe foil. Compared with Fe foil, the pre-edge of FePy@FePi and FePi shifted to higher energy position in XANES spectra. Additionally, the pre-edge peak of FePi is located at 7,134 eV, while the peak of FePy@FePi is located at 7,138 eV. This result fits well with the result showed in Fe 2*p* XPS spectra [44–46]. Because FePi amorphous layer contains Fe(II) spices, the Fe valance in FePi is a little bit lower than FePy@FePi. In the Fourier-transformed k^3^-weighted EXAFS curves (Fig. 2f), the peak at R space of around 1.5 Å can be attributed to Fe–P coordination in iron (pyro)phosphate groups, and the peak at around 1 Å in Fourier-transformed EXAFS curves relates to the nearest neighbor oxygen atoms [47, 48]. Importantly, the peaks intensity of FePy@FePi is higher than that of FePi, which is also related to the introduction of FePy crystalline phase [49]. According to the XANES and EXAFS results, we confirmed the unique heterostructure composed of crystalline FePy and amorphous FePi in the FePy@FePi hybrid overlayer.

**Fig. 3:**
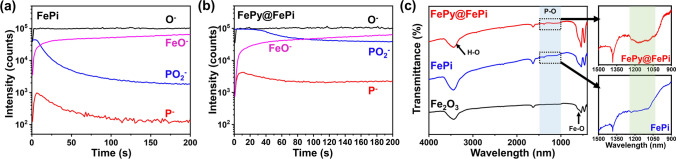
**a, b** ToF–SIMS depth profiling for negative ion polarity for FePi and FePy@FePi. **c** FT-IR spectra of α‐Fe_2_O_3_, FePi, and FePy@FePi

To complement the XPS results and further elucidate the difference between the FePy@FePi and FePi overlayers, time-of-flight secondary ion mass spectrometry (ToF–SIMS) depth profiles were measured (Fig. 3a-b). Molecular fragments including FeO^−^, PO_2_^−^, P^−^, and O^−^ ions are presented in Fig. 3a-b for FePi and FePy@FePi decorated photoanode samples, respectively. The signals of PO_2_^−^ and P^−^ are originated from the decomposition of the phosphate species. The intensities of PO_2_^−^ and P^−^ for FePi decorated photoanode were rapidly decreased as same depth profile. However, those for FePy@FePi decorated photoanode were much gradually decreased as time goes by, and PO_2_^−^ signal maintain its high intensity for much longer time in FePy@FePi. This indicates that different decomposition of the phosphate species in FePy@FePi and FePi overlayer, thereby they are composed of different kinds of phosphate species [50]. Noticeably, the absence of Fe^−^ fragments indicated the absence of iron phosphide species in these samples [51].

Fourier-transform infrared (FT-IR) spectroscopy was performed on the series of as-synthesized samples (Fig. 3c). Bands assigned to phosphate groups can be identified in the wavelength range between 1000 and 1500 cm^−1^ [52]. In the spectrum of FePy@FePi, a series of characteristic bands can be found in the wavelength range of 1000–1250 cm^−1^ while did not appear in the spectrum of FePi. These bands are attributed to the P-O bridging and linkage between phosphate groups, supporting the presence of pyrophosphate groups [52]. However, due to the relatively low content, peaks or bands in the range of 400–1000 cm^−1^ are difficult to identify. Additionally, there were no bands related to Fe–P bonding that were observed in FT-IR spectra, which also corroborates the absence of iron phosphide species in FePy@FePi and FePi.

**Fig. 4:**
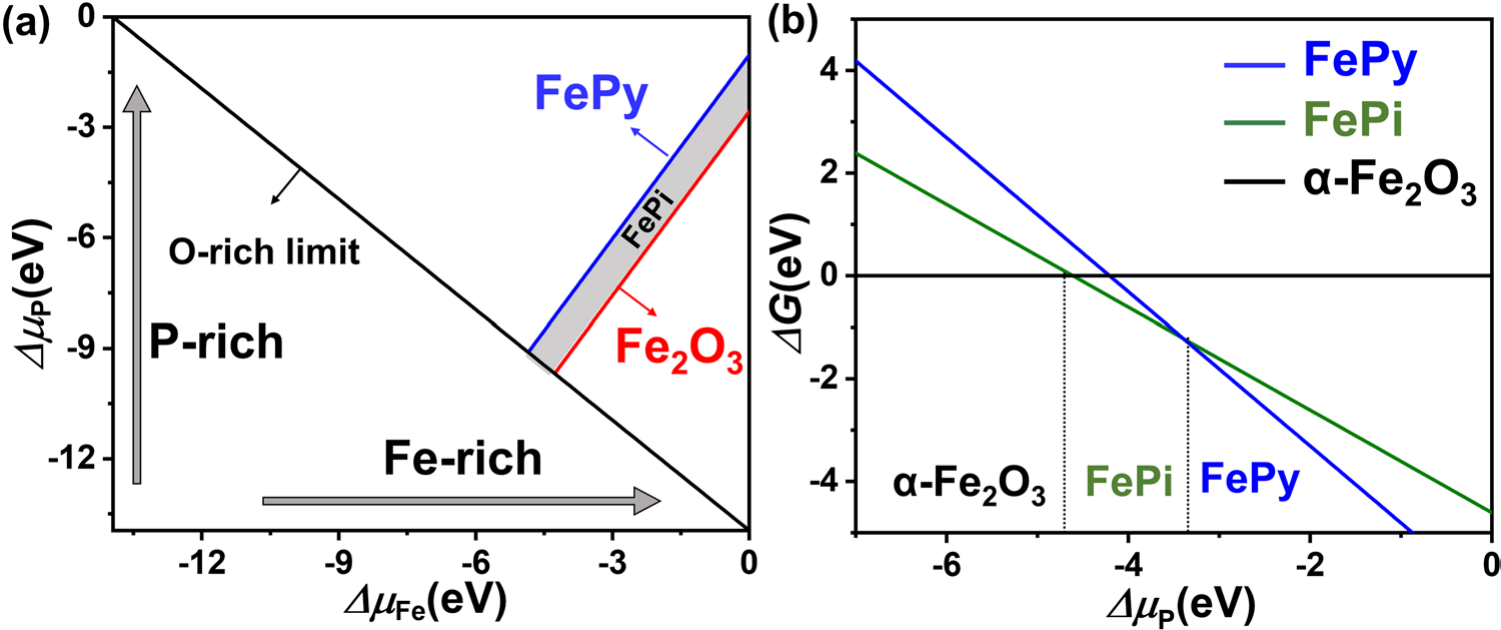
Theoretically prediction of phosphating process. **a** Phase space of FePy, FePi, and α-Fe_2_O_3_ under chemical potential variations of Fe, P, and O.** b** Gibbs free energy change for phase transformation from α-Fe_2_O_3_ to FePi and FePy

To fully illustrate the generation mechanism of FePy@FePi hybrid overlayer during the phosphating process, we theoretically verified the thermodynamic stability of the FePi and FePy phases produced in the α-Fe_2_O_3_ phosphating process through DFT-thermodynamic calculations (Fig. 4). The FePi phase formation in the presence of Fe_2_O_3_ by surface phosphating is:

1 $$ \Delta \mu_{{{\text{Fe}}}} + \Delta \mu_{P} + 4\Delta \mu_{O} = \Delta E_{f}^{{{\text{FePO}}_{{4}} }} $$

The chemical potential change of each element *i* by the formation of FePi from is defined as:

2 $$ \Delta \mu_{i} = \mu_{i} - \mu_{i}^{0} . $$

The $\mu_{i}^{0}$ term is the energy of element *i* in its pure state, such as metallic Fe, P, and gaseous O_2_. The phosphating process involves P vaporization at 300 °C. Therefore, the lower and upper limits of the phosphorus chemical potential, which are consistent with the extremely P-poor and P-rich conditions in the phosphating processes, are given by the following equations.

3 $$ \mu_{P }^{0} + \Delta E_{f}^{{{\text{FePO}}_{4} }} \le \mu_{P} \le \mu_{P}^{0} $$

4 $$ \mu_{P} \left( {T,P_{P} } \right) = \left\{ {\tilde{\mu }_{P} \left( {T,P^{^\circ } } \right) + k_{B} T\ln \left( {\frac{{P_{P} }}{{P^{^\circ } }}} \right)} \right\} $$

The $\mu_{P}^{0}$ term in Eqs. 3 and 4 is the chemical potential of phosphorus in the gas phase. The formation of *α-*Fe_2_O_3_ and FePy became spontaneous under the following conditions.

5 $$ 2\Delta \mu_{{{\text{Fe}}}} + 3\Delta \mu_{O} > \Delta E_{f}^{{{\text{Fe}}_{{2}} {\text{O}}_{{3}} }} $$

6 $$ 4\Delta \mu_{Fe} + 6\Delta \mu_{P} + 21\Delta \mu_{O} > \Delta E_{f}^{{{\text{Fe}}_{{4}} {\text{(P}}_{{2}} {\text{O}}_{{7}} {)}_{{3}} }} $$

The Inequality 5 and 6 are depicted in Fig. 4a, which shows that FePi and FePy become stable as Fe_2_O_3_ reacts with vaporized phosphorus. In the P-rich region, FePy becomes more stable than FePi. The reaction temperature (300 °C) of *α-*Fe_2_O_3_ with NaH_2_PO_2_·H_2_O in the reaction furnace is slightly below the melting point of NaH_2_PO_2_·H_2_O, which is 310 °C. Therefore, even the very low partial pressure of P at 300 °C can react to form FePi and FePy by the partial decomposition of NaH_2_PO_2_·H_2_O. As shown in Fig. 4b, FePi becomes the most stable in the range of − 4.62 eV < $\Delta \mu_{i}$ <  − 3.40 eV and FePy becomes the stable phase when $\Delta \mu_{i}$ > − 3.40 at 300 °C. At lower and higher temperatures than 300 °C, larger mole fractions of FePi and FePy phases are predicted from the theoretical Gibbs free energy change (Fig. 4b).

Thus, we can hypothesize the synthesis mechanism of the FePy@FePi hybrid overlayer. After the electrochemical treatment, FeOOH was produced on the surface of the α-Fe_2_O_3_ NRs and led to partial activation. In the activated regions, a hydrophobic surface can be formed. Because the phosphide species are difficult to adsorb on the hydrophobic surface, they will be enriched in the other regions [53–55]. In the P-rich region, nanocrystalline FePy is preferentially generated during the phosphating process, while the other regions are conducive to the production of amorphous FePi.

### Water Oxidation Performance

**Fig. 5:**
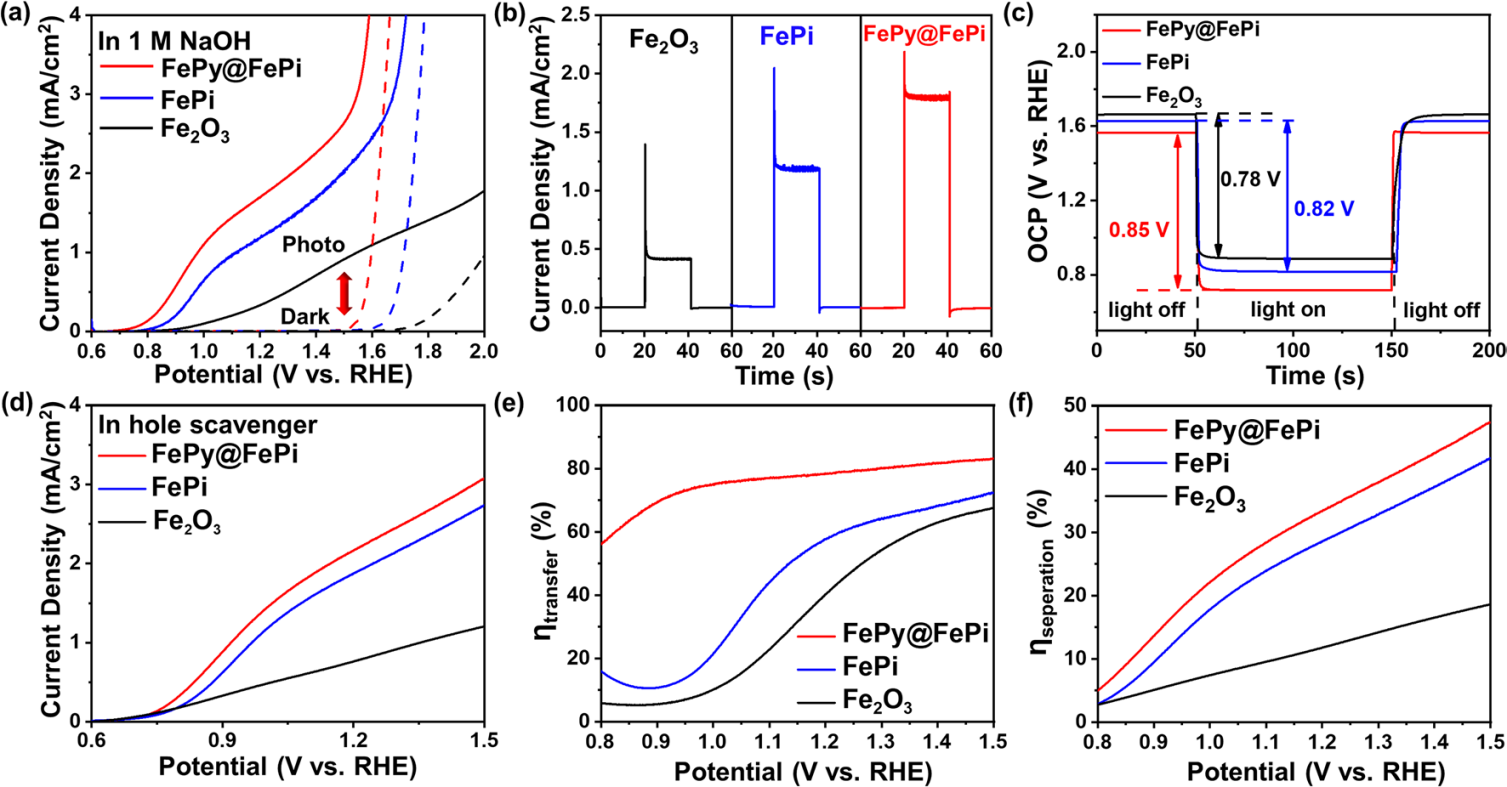
PEC performance of as-synthesized samples. **a** Current density versus potential RHE (*J-V)* curves under dark and 1 sun illumination in 1 M NaOH electrolyte. **b** Chopped current density versus time *(J-t)* curves of Fe_2_O_3_, and FePi, FePy@FePi decorated photoanodes under dark and 1 sun illumination conditions in 1 M NaOH electrolyte at 1.23 V versus RHE. **c** Open circuit potential curves under chopped light. **d** *J-V* curves measured with the hole scavenger. **e** Charge transfer efficiency (*η*_*transfer*_). **f** Charge separation efficiency (*ηs*_*eparation*_) versus RHE curves of fabricated photoanodes

The water splitting performance of the as-synthesized photoanode was investigated by performing a linear sweep voltammetry (LSV) test. Figure 5a shows the *J-V* curves of the photoanodes tested under AM 1.5G 1 sun illumination (100 mW cm^−2^). At a potential of 1.23 V versus RHE in 1 M NaOH electrolyte, the pristine α-Fe_2_O_3_ photoanode yields a photocurrent density of 0.36 mA cm^−2^. Increases to 1.18 mA cm^−2^ for the FePi decorated photoanode and 1.78 mA cm^−2^ for FePy@FePi decorated photoanode were obtained, which corresponds to enhancements of 3.23- and 4.89-folds, respectively. FePy@FePi hybrid overlayer demonstrates much more significant improvement than FePi single-phase overlayer. Additionally, the onset potential of the *J-V* curve for FePy@FePi measured under dark conditions was dramatically reduced compared to those of pristine α-Fe_2_O_3_ and FePi, which shows an improved capability for electrochemical water oxidation. To further analyze the charge transfer characteristic of these samples, the photocurrent transient curves of α-Fe_2_O_3_, FePi, and FePy@FePi decorated photoanodes were measured at 1.23 V versus RHE in 1 M NaOH electrolyte (Fig. 5b). All of the samples show negative and positive photocurrent spikes. The positive spike is related to the accumulation of holes of the photoanode under illumination, while the negative spike is attributed to the recombination of the bulk electrons and the accumulated holes during illumination. The average photocurrent decays of the α-Fe_2_O_3_, and FePi, FePy@FePi decorated photoanodes were calculated to be 0.98, 0.87, and 0.39 mA cm^−2^, respectively. These results indicate that FePy@FePi hybrid overlayer is more effective in inhibiting the electron hole recombination of the α-Fe_2_O_3_ photoanode compared to the homogenous FePi amorphous overlayer, which is preferred to achieve a higher charge transfer efficiency. In Fig. 5c, the transient open circuit potential (OCP) under illumination and dark condition was measured to study the charge recombination of these samples. For all these samples, the decrease of open circuit potential upon illumination relates to electron–hole pair generation. Compared with α-Fe_2_O_3_ (Δ_OCP_ = 0.78 V) and FePi (Δ_OCP_ = 0.82 V), FePy@FePi (Δ_OCP_ = 0.85 V) shows the largest OCP difference. This indicates that the hybrid FePy@FePi overlayer is more effective in enhancing electron–hole pair separation and suppressing recombination than the amorphous FePi overlayer. The change of photovoltage is mainly related to the trap states generated after the surface-combined (pyro) phosphate overlayer. Surface trap states lead to Fermi level pinning in the space-charge region, limiting the energy barrier height, which is generally considered to be the direct cause of the drop in photovoltage [56]. Furthermore, the LSV test in the presence of a hole scavenger (Na_2_SO_3_) is further characterized (Fig. 5d**)**. The absorptance spectra (Fig. S7a) were obtained by considering the UV–Vis diffuse reflectance (*R*) and diffuse transmittance (*T*) using the equation *A* = 100% − *R* (%)–*T* (%). Based on the LSV results (Fig. 5a, d**)**, the charge transfer efficiency (*η*_transfer_) was calculated (*η*_transfer_ = *J*_water_*/J*_scavenger_) and is displayed in Fig. 5e. The *η*_transfer_ values at 1.23 V versus RHE were 45% for pristine α-Fe_2_O_3_, 60% for FePi, and 79% for FePy@FePi. The significantly high *η*_transfer_ demonstrates that the incorporation of the FePy crystalline phase in the surface amorphous overlayer efficiently suppresses the surface charge recombination and accelerates the water oxidation kinetics. Furthermore, based on the absorption photocurrent density (*J*_abs_) and *J*_scavenger_, the charge separation efficiency of the photoelectrode can be calculated (*ηs*_eparation_ = *J*_scavenger_*/J*_abs_), as shown in Fig. 5f. The charge separation efficiency at 1.23 V versus RHE of pristine α-Fe_2_O_3_ was calculated to be 12% and this value further increased to 30% (FePi) and 35% (FePy@FePi), which quantitatively shows the promoting effect of FePy nanocrystalline phase decorating on the charge separation efficiency.

**Fig. 6:**
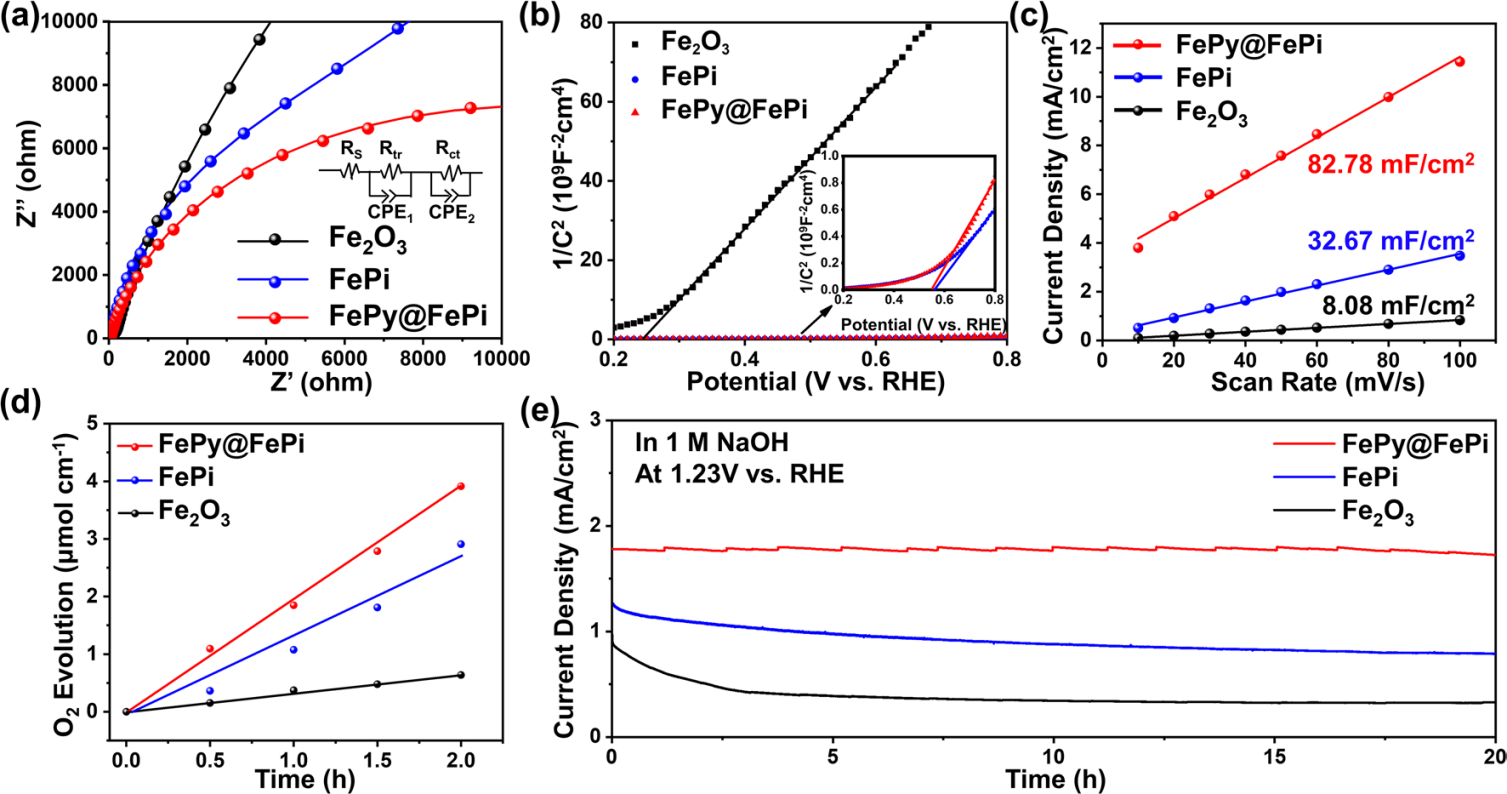
**a** Nyquist plots from EIS characterization under 1 sun illumination in 1 M NaOH electrolyte at 1.23 V versus RHE.** b** Mott–Schottky plots of fabricated photoanodes under dark conditions in 1 M NaOH electrolyte. **c** Electrochemical double-layer capacity (*C*_dl_) of FePy@FePi, FePi, and Fe_2_O_3_. **d** O_2_ evolution-time plot of fabricated photoanodes. **e** *J-t* curves of the fabricated photoanodes under 1 sun illumination conditions in 1 M NaOH electrolyte at 1.23 V versus RHE for a 20 h stability test

To further investigate the catalytic mechanism of the hybrid overlayer, electrochemical impedance spectroscopy (EIS) was carried out at an open circuit potential under 1 sun illumination. As shown in Fig. 6a, a significant decrease of the charge transport resistance in the bulk (*R*_tr_) was observed on the FePi (6.675 kΩ) and FePy@FePi decorated photoanodes (2.177 kΩ), indicating that the surface overlayer can effectively improve the charge separation efficiency. Meanwhile, the resistance of charge transfer across the electrode/electrolyte (*R*_ct_) also decreased from 56.77 kΩ for Fe_2_O_3_ to 31.9 kΩ for FePi and further decreased to 17.92 kΩ for the FePy@FePi decorated photoanode, proving that the surface charge transfer is promoted by incorporating the FePy nanocrystalline phase in the amorphous overlayer. As a reference, series PEC performance characterizations were also conducted on an electrochemically treated α-Fe_2_O_3_ photoanode. The variation of the charge transfer/transport resistance are consistent with the trends of the donor density and flat band potential value obtained by the Mott–Schottky analysis **(**Fig. 6b). This indicates pronounced Fermi level pinning due to passivated surface states, which also decreased surface trap sites and suppressed electron–hole recombination process [57]. Additionally, the experimental bandgap of α-Fe_2_O_3_, FePi on α-Fe_2_O_3_, and FePy@FePi on α-Fe_2_O_3_ samples was presented in Fig. S7b. The similar bandgaps of FePy@FePi on α-Fe_2_O_3_ (ca. 2.04 eV) and FePi on α-Fe_2_O_3_ (ca. 2.04 eV) reveal that the effect of their light absorption property on PEC performance is negligible. Furthermore, the electrochemically active surface area (ECSA) of FePy@FePi is higher than that of FePi and α-Fe_2_O_3_, indicates the hybrid overlayer providing more active site for the PEC water oxidation reaction (Figs. 6c and S8). The gas production rate is evaluated by gas chromatography system. FePi and FePy@FePi decorated photoanode produced 1.07 and 1.85 µmol h^−1^ cm^−2^ of O_2_, respectively (Fig. 6d). FePy@FePi decorated photoanode shows a Faradaic efficiency at 65.86% for O_2_ production after 1 h is also higher than that of FePi (Fig. S9).

The long-term stability of the FePy@FePi decorated photoanode was measured for over 20 h, where the current–time curve demonstrates that the FePy@FePi hybrid overlayer has good stability under the experimental conditions (Fig. 6e). In contrast, the long-term stability of FePi overlayer and α-Fe_2_O_3_ is unsatisfactory. As shown in the HRTEM images (Fig. S10), the nanocrystalline phase of FePy maintains its high crystallinity after the PEC oxygen evolution reaction, and the FePy@FePi hybrid overlayer was still conformally covered on the surface of α-Fe_2_O_3_ nanorods without decomposition. The XPS results of the FePy@FePi decorated photoanode after the stability test are shown in Fig. S11. The deconvoluted peak belongs to P-O bond still shows high intensity, which also confirms that the FePy@FePi overlayer is stable in the photoelectrochemical water oxidation reaction. A sodium Auger peak (Na KLL) is observed in the O 1* s* spectrum at around 535 eV, which is due to the surface-absorbed electrolyte during the electrochemical measurement. According to the HRTEM images and XPS spectra after the long-term stability test, the FePy@FePi overlayer was stable in both morphology and composition. The complete co-catalyst overlayer can protect the α-Fe_2_O_3_ matrix from photocorrosion by preventing the direct contact of the electrolyte with the metal oxide matrix, thereby improving the overall stability [58].

### DFT Calculation

**Fig. 7:**
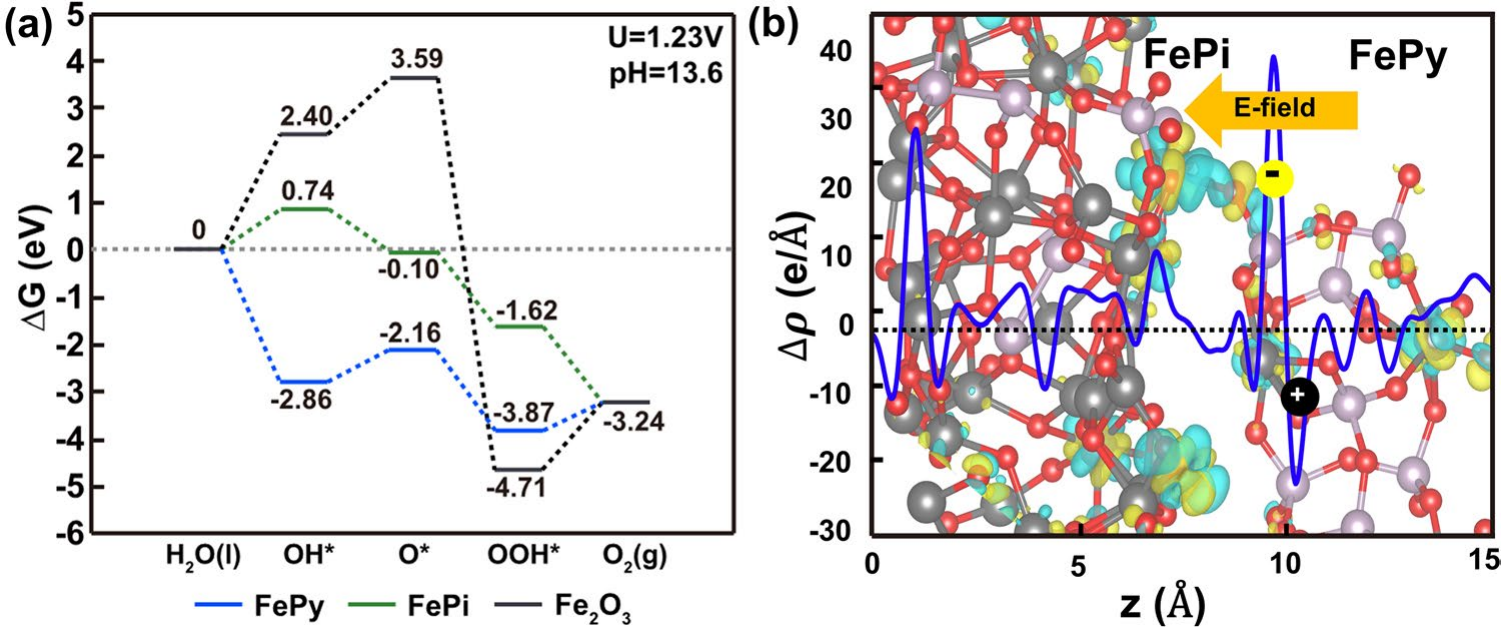
**a** Adsorption free energy diagrams of OER on crystalline FePy, FePi, and *α-*Fe_2_O_3_ (0 1 2) surface under the conditions of *U *= 1.23 V versus RHE and pH = 13.6 from DFT. **b** The plane-averaged differential charge densities at the FePi/FePy interface

To further understand the role of this hybrid overlayer with nanocrystalline FePy and amorphous FePi in the water oxidation reaction, DFT calculations were conducted to theoretically analyze the surface reaction process. Our DFT calculations show that coexistence of FePi and FePy phases on the surface results in much better OER kinetics under *U* = 1.23 V and pH = 13.6 conditions, as shown in Fig. 7a. In our hypothesized reaction model, all of iron atoms in FePy, FePi, and α-Fe_2_O_3_ phase were active sites in PEC oxygen evolution reaction. The FePi was found to have a typical down-hill reaction for the conversion from OH* to O_2_, while the up-hill energy for the conversion of H_2_O to OH* limited the water adsorption efficiency on the FePi phase. On the other hand, FePy has a low free energy (− 2.86 eV) for the formation of OH* in the exothermic reaction, which is even better than a previously reported crystalline metal phosphate [59]. However, the endothermic reactions in the conversion from OH* to O_2_ on FePy result in up-hill energy barriers of 0.70 eV for O* and 0.63 eV for O_2_. Therefore, the FePy and FePi hybrid can further improve the overall OER activity via the intermediate exchange between the FePy and FePi phases. In recent theoretical and experimental studies, alternating reaction pathways in heterogeneous electrocatalysts have been well demonstrated [60, 61]. Based on diffusion theory, the surface diffusion and adsorbate exchange of the OH* species between FePy and FePi phases is driven by concentration gradients. The improved OER activity by the formation of the FePy phase on FePi is consistent with our experimental results of a larger current density of FePy@FePi than that of FePi decorated photoanode. The overall OER on the Fe_2_O_3_ (012) surface is significantly inferior compared to FePi and FePy. The calculated plane-averaged charge density by FePi/FePy heterojunction formation shows that an interface dipole moment by charge transfer from FePi to FePy, a built-in electric field directed from FePy to FePi, is induced (Fig. 7b). Therefore, photoexcited hole carriers are delivered from FePy to FePi effectively. From the DFT calculation results, we can reason that FePy phase formation can provide at least two advantages of PEC water splitting by the FePi surface: i) stabilization of OH^−^ on the surface and ii) hole delivery for further oxidation reactions.

## Conclusions

In summary, we demonstrated a strategy to localize the crystalline FePy in the amorphous iron phosphate-based overlayer for boosting PEC water oxidation. The phosphate-based overlayer with a heterogeneous hybrid structure was regulated by incorporating FePy, which resulted in significantly enhanced PEC performance. Around a five folders higher photocurrent density was obtained by FePy@FePi decoration (1.78 mA cm^−2^), compared to that of pristine α-Fe_2_O_3_ (0.36 mA cm^−2^) at 1.23 V versus RHE under 1 sun illumination. The charge transfer efficiency was significantly enhanced to 79% after decorating FePy@FePi hybrid overlayer. Additionally, the FePy@FePi hybrid overlayer achieved long-term durability over 20 h under at 1.23 V versus RHE under 1 sun illumination. DFT calculations revealed that the heterogeneous hybrid structure overcomes the energy barrier for water absorption and transition. All of these results demonstrate that the FePy@FePi hybrid overlayer with phosphate played a key role in accelerating the surface PEC water oxidation kinetics in addition to passivating the surface trap states. We believe that this work introduces a new strategy to introduce novel hybrid structures composed of nanocrystalline phases into the surface overlayer, providing inspiration for the synthesis of high-efficiency water oxidation photoanodes.

## Supplementary Information

Below is the link to the electronic supplementary material.

Supplementary file1 (PDF 1357 KB)
