# Supplementary material for: Nanocrystalline Iron Pyrophosphate-Regulated Amorphous Phosphate Overlayer for Enhancing Solar Water Oxidation
Source: Nanomicro Lett. 2022 Oct 31;14:209. doi: 10.1007/s40820-022-00955-w (PMC9622969; doi:10.1007/s40820-022-00955-w)
Supplement: Supplementary file 1 — Supplementary file1 (PDF 1357 KB) [file 40820_2022_955_MOESM1_ESM.pdf]

Supporting Information for

Nanocrystalline Iron Pyrophosphate Regulated Amorphous Phosphate Overlayer for Enhancing Solar Water Oxidation

Chengkai Xia[1], Yuankai Li[1], Minyeong Je[2], Jaekyum Kim[1], Sung Min Cho[1], Chang Hyuck Choi[3], Heechae Choi[2], Tae-Hoon Kim[4], and Jung Kyu Kim[1,*]

[1]School of Chemical Engineering, Sungkyunkwan University (SKKU), 2066 Seobu-ro, Jangan-gu, Suwon, 16419, Republic of Korea

[2]Theoretical Materials & Chemistry Group, Institute of Inorganic Chemistry, University of Cologne, Greinstr. 6, 50939, Cologne, Germany

[3]Department of Chemistry, Pohang University of Science and Technology (POSTECH), Pohang, 37673, Republic of Korea

[4]Department of Materials Science & Engineering, Engineering Research Center, Chonnam National University, Gwangju, 61186, Republic of Korea

*Corresponding author. E-mail: legkim@skku.edu (Jung Kyu Kim)

S1 Experimental Detail

S1.1 Chemicals

All materials generated in this study are available from the lead contact without restriction. Iron (III) chloride hexahydrate ($FeCl_3 \cdot 6H_2O$; Sigma-Aldrich; AR 97.0%), urea ($(NH_2)_2CO$; Sigma-Aldrich; AR, 99%), hydrochloric acid (HCl; Sigma-Aldrich; 36.5-38.0%), sodium hypophosphite monohydrate ($NaH_2PO_2 \cdot H_2O$; AR, 99%), sodium hydroxide (NaOH; Sigma-Aldrich; AR, > 85%), sodium sulfite (Na_2SO_3; AR 97.0%), and fluorine-doped tin oxide glass (FTO, 15 Ω, thickness 2 mm) were used as received unless stated otherwise.

S1.2 Faradic Efficiency Calculation

The faradaic efficiency of O_2 production was calculated using the follow equation:

$$Faradaic\ efficiency = nO_2/(Q/2F) \qquad \text{(S1)}$$

where nO_2 defines produced oxygen with the utilization of total charge Q, F refers to Faradic constant.

S1.3 DFT Calculations

DFT calculations were performed using the Vienna *ab initio* package (VASP) [S1, S2]. The core electrons were described by using the projector augmented wave (PAW) method [S3]. A generalized gradient approximation (GGA) with the Perdew-Burke-Ernzerhof (PBE) functional was employed for the plane-basis wave expansion [S4, S5]. A corrective Hubbard U correction (GGA+U) method introduced by Dudarev *et al.* was included in the calculations to accurately describe the strongly correlated interaction of Fe 3d orbitals [S6]. U_{eff} was set to 5 eV for Fe atoms. The energy cut-off of 400 eV was used. Brillouin zones were U_{eff} was set to 5 eV for Fe atoms and an energy cut-off of 400 eV was used. Brillouin zones were sampled with a gamma-centered k-point grid of 1 × 2 × 1 in the supercell of FePy, FePi, and α-Fe_2O_3(012) of which the lateral dimensions are 21.71 × 9.68 Å^2, 18.24 × 18.22 Å^2, and 19.39 × 10.28 Å^2, respectively [S7]. Large enough vacuum layers of at least 15 Å were applied to minimize undesirable interactions between adjacent periodic cells. The Gaussian smearing scheme was applied with

a smearing width of 0.1 eV. The energy convergence criteria in the self-consistent field were set to 10^{-6} eV and geometry structures were fully relaxed with Hellman-Feynman forces with a tolerance of 0.1 eV $Å^{-1}$.

The ΔG of the OER intermediates was defined as follows:

$$\Delta G = \Delta E + \Delta ZPE - T\Delta S + \Delta G_U + \Delta G_{pH} \quad \text{(S2)}$$

where ΔE is the calculated total energy difference, ΔZPE and $T\Delta S$ are the zero-point energy correction and entropy terms, respectively, which can be determined by frequency calculations. ΔG_{pH} is the correction of the H^+ free energy by the concentration and ΔG_U represents the free energy terms related to the applied electrode potential, U.

S2 Supplementary Figures

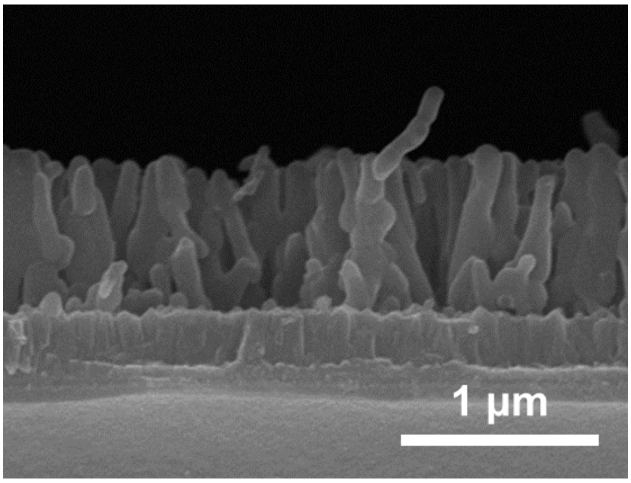

Fig. S1 Cross slide SEM image of FePy@FePi decorated nanorods

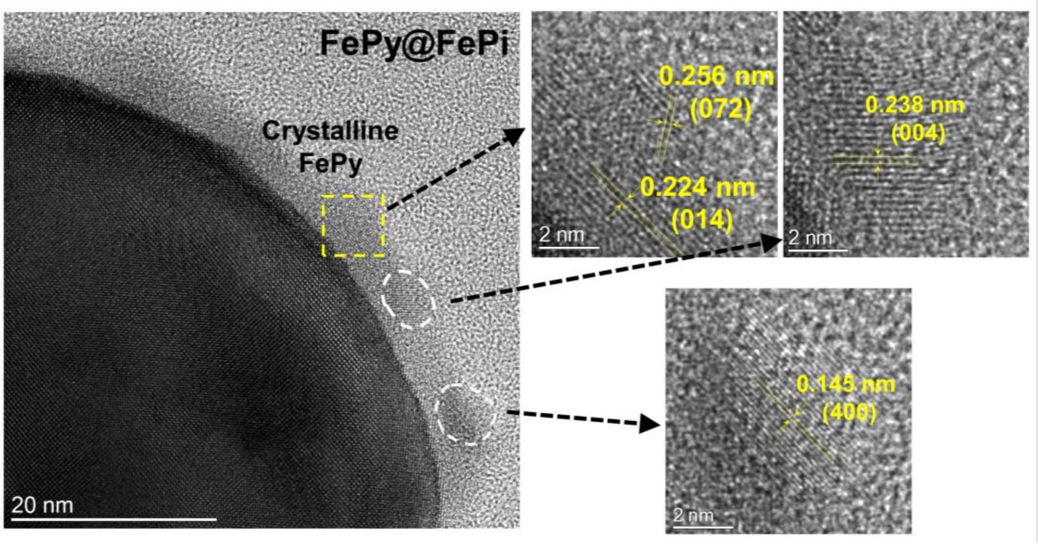

Fig. S2 HRTEM image of FePy@FePi hybrid overlayer and lattice fringe of $Fe_4(P_2O_7)_3$ crystalline phase

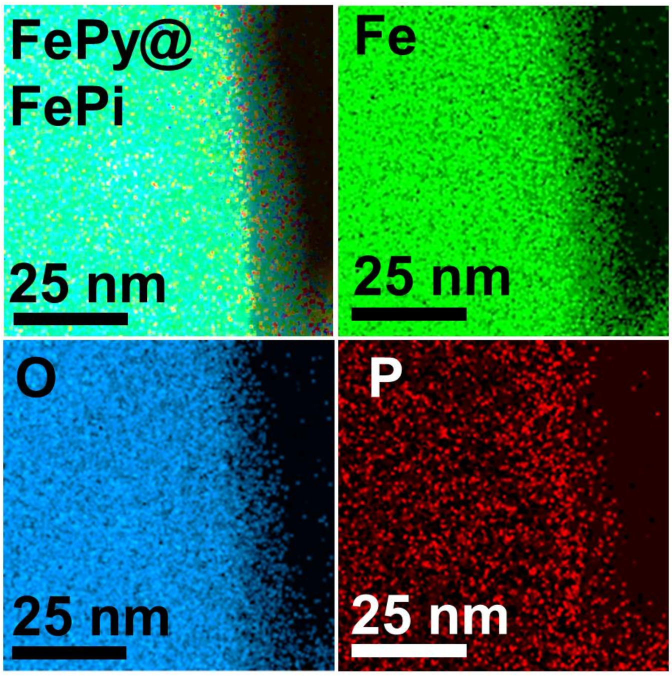

Fig. S3 EDX images of the FePy@FePi hybrid overlayer

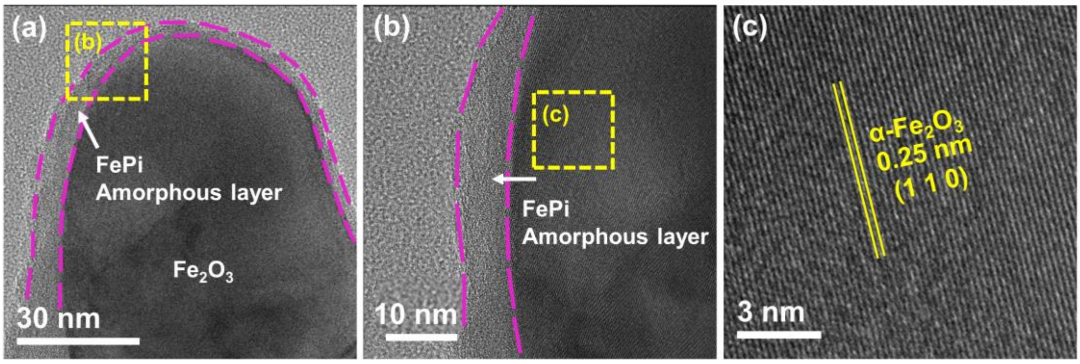

Fig. S4 a-c HR-TEM images of FePi at different magnifications

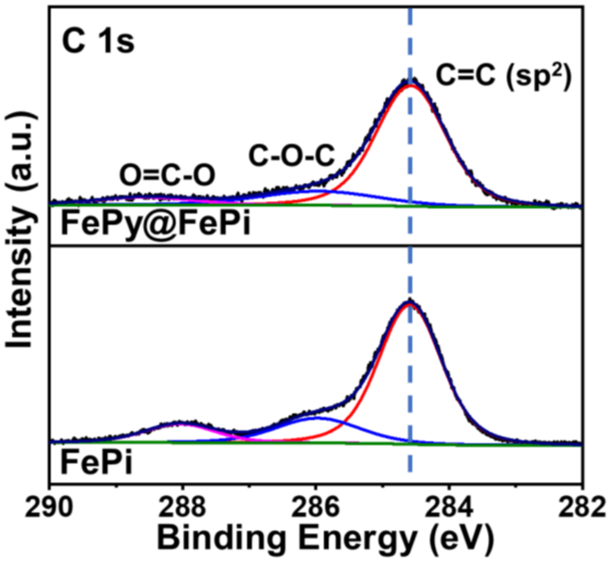

Fig. S5 XPS spectra of C 1*s* for FePi, and FePy@FePi

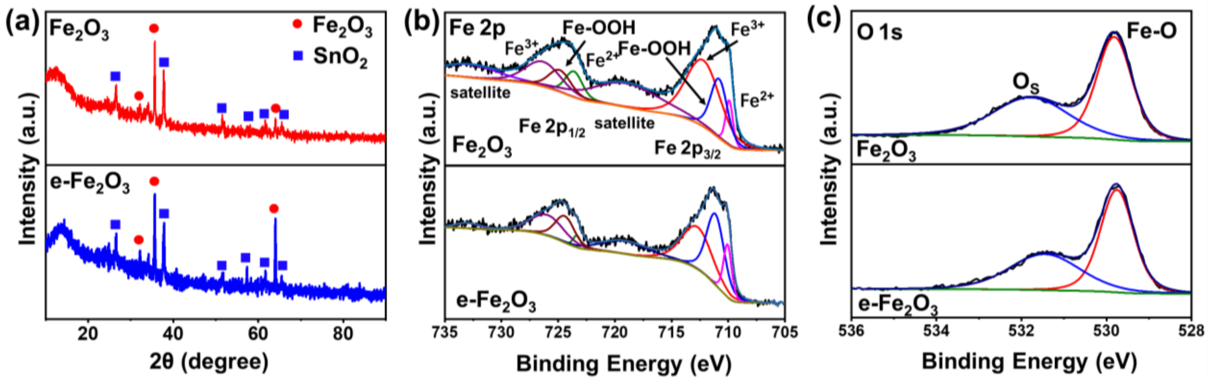

Fig. S6 a XRD patterns of Fe_2O_3 and e-Fe_2O_3. **b, c** XPS spectra of Fe 2p and O 1s of Fe_2O_3 and e-Fe_2O_3

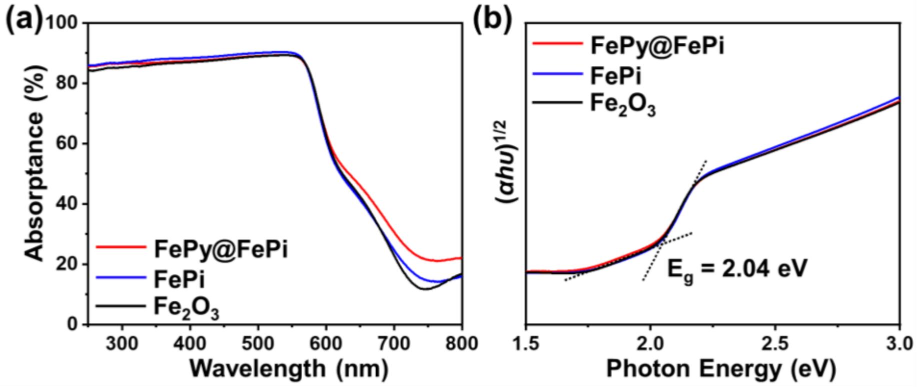

Fig. S7 a UV-vis absorptance spectra of Fe_2O_3, FePi, and FePy@FePi decorated photoanode. The absorptance spectra (A) were obtained by considering the diffuse reflectance (R) and diffuse transmittance (T) spectra (A = 100 % – R – T). **b** Tauc plots of α-Fe_2O_3 and FePi, and FePy@FePi decorated α-Fe_2O_3

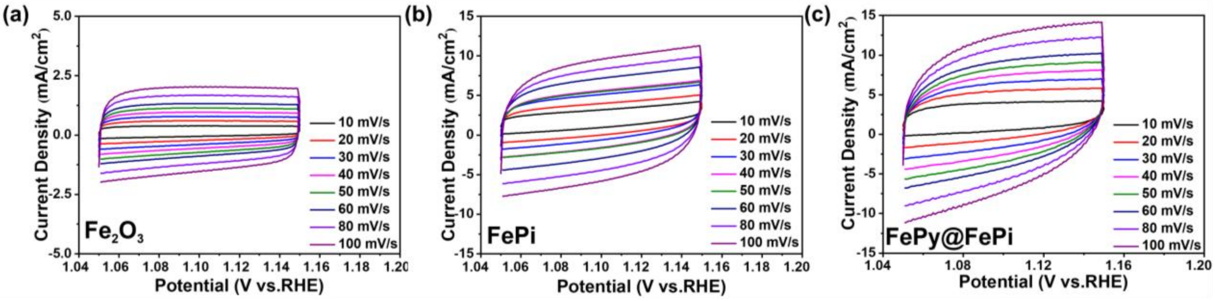

Fig. S8 a-c Cyclic voltammetry curves of Fe_2O_3, FePi, and FePy@FePi decorated photoanode

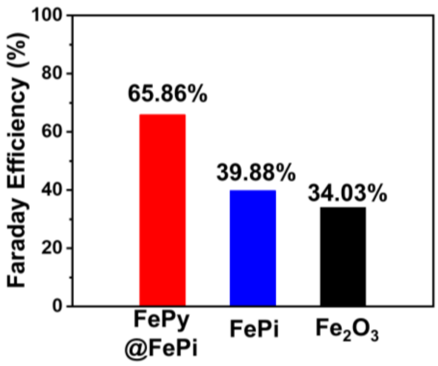

Fig. S9 Faradic Efficiency of Fe_2O_3, FePi, and FePy@FePi decorated photoanode measured at 1.23 V vs. RHE at 1 sun illumination after measured for 1 h

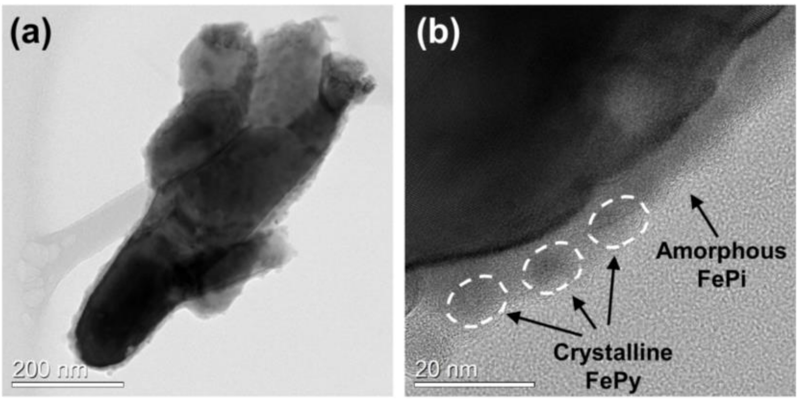

Fig. S10 a HRTEM image of FePy@FePi hybrid overlayer decorated α-Fe_2O_3 nanorod after PEC test. **b** HRTEM image of FePy@FePi hybrid overlayer after PEC test

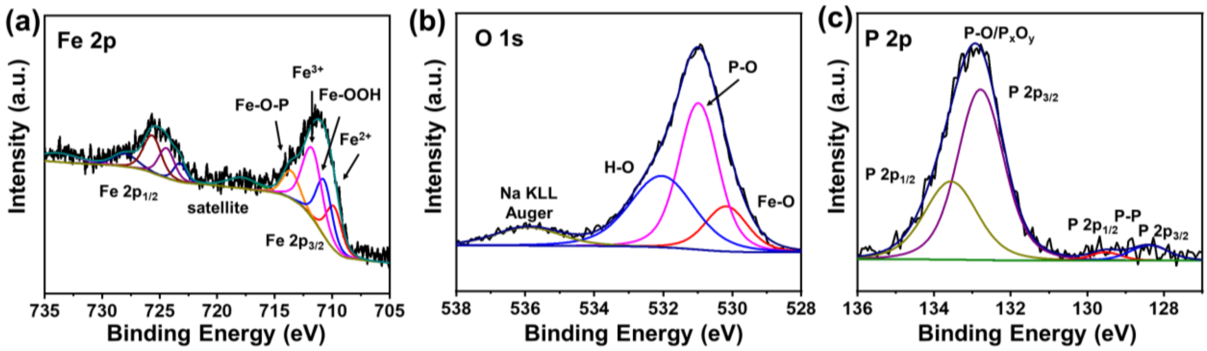

Fig. S11 a-c Fe 2p, O 1s, and P 2p XPS spectra of FePy@FePi decorated photoanode after stability test

Supplementary References

[S1] G. Kresse, J. Furthmüller, Efficiency of ab-initio total energy calculations for metals and semiconductors using a plane-wave basis set. Comput. Mater. Sci. **6**(1), 15-50 (1996). https://doi.org/10.1016/0927-0256(96)00008-0

[S2] G. Kresse, J. Furthmüller, Efficient iterative schemes for ab initio total-energy calculations using a plane-wave basis set. Phys. Rev. B **54**(16), 11169 (1996). https://doi.org/10.1103/PhysRevB.54.11169

[S3] G. Kresse, D. Joubert, From ultrasoft pseudopotentials to the projector augmented-wave method. Phys. Rev. B **59**(3), 1758 (1999). https://doi.org/10.1103/PhysRevB.59.1758

[S4] J.P. Perdew, J.A. Chevary, S.H. Vosko, K.A. Jackson, M.R. Pederson et al., Atoms, molecules, solids, and surfaces: applications of the generalized gradient approximation for exchange and correlation. Phys. Rev. B **46**(11), 6671 (1992). https://doi.org/10.1103/PhysRevB.46.6671

[S5] J.P. Perdew, K. Burke, M. Ernzerhof, Generalized gradient approximation made simple. Phys. Rev. Lett. **77**(18), 3865 (1996). https://doi.org/10.1103/PhysRevLett.77.3865

[S6] S.L. Dudarev, G.A. Botton, S.Y. Savrasov, C.J. Humphreys, A.P. Sutton, Electron-energy-loss spectra and the structural stability of nickel oxide: an LSDA+U study. Phys. Rev. B **57**(3), 1505-1509 (1998). https://doi.org/10.1103/PhysRevB.57.1505

[S7] H.J. Monkhorst, J.D. Pack, Special points for brillouin-zone integrations. Phys. Rev. B **13**(12), 5188 (1976). https://doi.org/10.1103/PhysRevB.13.5188
